# The complete chloroplast genome of *Camellia grijsii*, an ornamental shrub with floral aroma

**DOI:** 10.1080/23802359.2021.1875901

**Published:** 2021-03-11

**Authors:** Fengyu Xie, Yingkun Sun, Xinlei Li, Ziyan Nie, Hengfu Yin, Jiyuan Li

**Affiliations:** a College of Landscape and Forestry, Qingdao Agricultural University, Qingdao, China; b State Key Laboratory of Tree Genetics and Breeding, Research Institute of Subtropical Forestry, Chinese Academy of Forestry, Hangzhou, China

**Keywords:** *Camellia grijsii*, chloroplast genome, phylogenetic analysis

## Abstract

*Camellia grijsii* is an ornamental shrub with a floral aroma, which is widely cultivated and used for landscaping in China. To obtain the genetic information of *C. grijsii*, we have sequenced and assembled the complete chloroplast (cp) genome based on the Illumina Hiseq platform. The total genome size is 161,078 bp in length with 37.18% GC, which contains a large single copy (LSC, 84,645 bp) region, a small single copy (SSC, 15,772 bp) region, and a pair of inverted repeat (IRs, 30,330 bp) regions. It is composed of 81 protein-coding genes, 4 ribosomal RNAs, and 43 transfer RNAs. The cp genome of *C. grijsii* has also been compared with other species of *Camellia*, and the results showed that the *C. grijsii* and the *C. grandbibracteata* are closely related. This study provides the complete cp genome of *C. grijsii* and has an important reference value for the evolutionary analysis.

*Camellia grijsii*, bearing dense white flowers and unique aroma, is an ornamental shrub. *C. grijsii* is mainly distributed in Fujian and Hubei province, and it is widely cultivated and used for landscaping in China. At present, the genomic information of the species is scarce. Compared with the nuclear genome and mitochondrial genome, the chloroplast genome contains the high conservation of sequences and is extensively and effectively used for genetic diversity and phylogenomic analyses. Also, a large amount of cp DNA markers have been selected for the utility of phylogeny or DNA barcoding (Asaf et al. 2017). In this study, we characterized the complete cp genome of *C. grijsii* (NCBI Accession Number: MT726053) by high-throughput sequencing. We described the assembly and annotation details of the cp genome and inferred the phylogeny of related *Camellia* species based on the whole cp genomes.

Fresh leaves of *C. grijsii* were collected in the *Camellia* Germplasm Resource Bank, Research Institute of Subtropical Forestry, Chinese Academy of Forestry (Hangzhou, Zhejiang, China; Coordinates: 119°95′E, 30°07′N). Total DNA was extracted using the MiniBEST plant Genomic DNA Extraction Kit (Takara, Dalian, China). The quantity of total DNA was higher than 20 ng/µL (total mass was higher than 100 ng) assessed by a NanoDrop2000 device (Thermo Fisher Scientific, USA). After DNA separation, the TruSeq DNA sample preparation kit (Illumina, San Diego, California, USA) was used to construct an Illumina library, and sequencing was performed by Illumina HiSeq 4000 platform (Illumina, San Diego, CA, USA) of Genesky Biotechnologies in Shanghai, China.

In total, 32,199,730 reads and 4,575,940,546 bases were gained at the beginning, then 30,392,793 clean reads and 4,415,577,651 clean bases were retrieved by quality control using Trimmomatic (Wang et al. 2020). The clean reads were firstly aligned to the reference genome sequence of *C. japonica* (NCBI Accession Number: NC_036830.1) through Bowtie v2.2.6 (Wang et al. 2018; Cao et al. 2020). The method of assembly and annotation of the cp genome was adapted from Wang et al. (2018). The sequence of cp genome was assembled using Newbler v3.0 (Ye et al. 2017) with the default parameters to predict protein-coding genes, ribosomal RNA (rRNA) genes and transfer RNA (tRNA) genes. Finally, manually confirmed by comparison with the chloroplast genome of C. japonica.

The length of complete cp genome of *C. grijsii*, with 37.18% GC, was 161,078 bp. It has a typical quadripartite organization, including of a large single-copy (LSC) region of 84,645 bp, a small single-copy (SSC) region of 15,772 bp, and a pair of inverted repeats (IRs) of 30,330 bp. There were 128 functional genes that included 81 protein-coding genes, 4 ribosomal RNAs, and 43 transfer RNAs.

Seventeen complete cp genomes of *Camellia* species were used for phylogenetic analyses. The conserved protein sequences were extracted for alignment (Wang et al. 2018), and the phylogenetic relationships were determined by MEGA v7.0.14 with the neighbor-joining method (Kumar et al. 2016). According to the phylogenetic tree, *C. grijsii* is evolutionally related to *C. grandbibracteata* (Figure 1).

**Figure 1.**
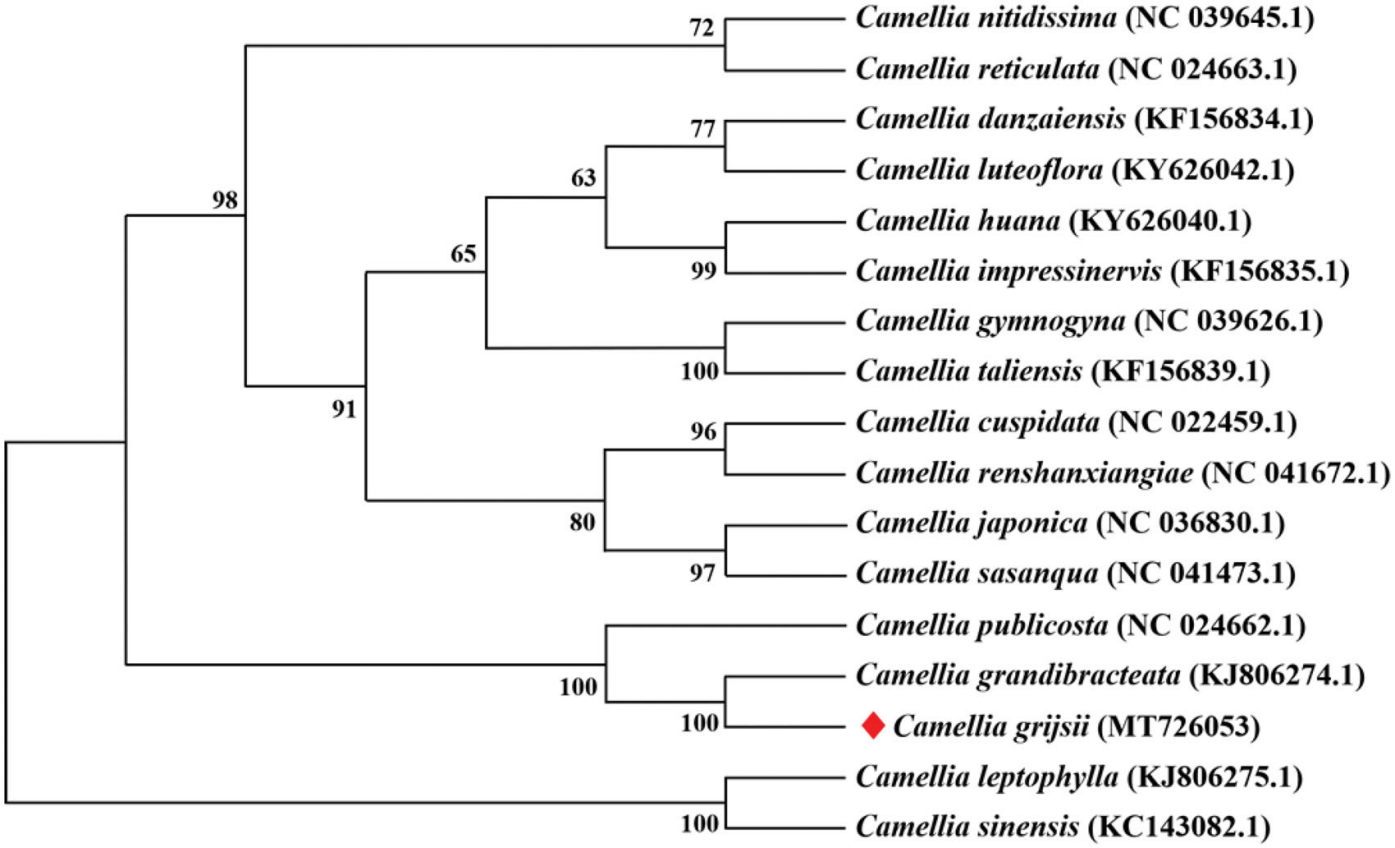
Neighbor-joining phylogenetic tree for *C. grijsii* with other *Camellia* species based on conserved protein sequences of cp genomes.

## Data Availability

The genome sequence data that support the findings of this study are openly available in GenBank of NCBI at https://www.ncbi.nlm.nih.gov under the accession no. MT726053 or from the corresponding author.
